# Examining the Influence of a New Light Rail Line on the Health of a Demographically Diverse and Understudied Population within the Washington, D.C. Metropolitan Area: A Protocol for a Natural Experiment Study

**DOI:** 10.3390/ijerph15020333

**Published:** 2018-02-13

**Authors:** Jennifer D. Roberts, Ming Hu, Brit Irene Saksvig, Micah L. Brachman, Casey P. Durand

**Affiliations:** 1 Department of Kinesiology, School of Public Health, University of Maryland, College Park, MD 20742, USA; 2 School of Architecture, Planning & Preservation, University of Maryland, College Park, MD 20742, USA; mhu2008@umd.edu; 3 Department of Epidemiology and Biostatistics, School of Public Health, University of Maryland, College Park, MD 20742, USA; bsaksvig@umd.edu; 4 Center for Geospatial Information Science, Department of Geographical Sciences, University of Maryland, College Park, MD 20742, USA; brachman@umd.edu; 5 Health Promotion and Behavioral Sciences, School of Public Health, The University of Texas Health Sciences Center, Houston, TX 77030, USA; casey.p.durand@uth.tmc.edu

**Keywords:** active transportation, light rail, built environment, natural experiment, sense of community

## Abstract

Approximately two-thirds of adults and youth in Prince George’s County, Maryland, a suburb of Washington, D.C. are overweight or obese and less than half are achieving daily physical activity recommendations. Active transportation (AT), such as walking, biking or using public transportation (PT), is a strategic pathway to improving physical activity levels and thus reducing excess weight. Utilizing an expansion of the Washington, D.C. area transportation system with a new light rail line, the Purple Line Outcomes on Transportation (PLOT) Study will exam pre- and post-Purple Line PT use, AT behaviors and attitudes and physical activity among Prince George’s County adults and youth. The PLOT Study will take advantage of this natural experiment in an area enduring significant racial/ethnic and gender-based overweight or obesity and physical inactivity disparities. While similar natural experiments on AT have been conducted in other U.S. cities, those studies lacked diverse and representative samples. To effectively evaluate these physical activity outcomes among this population, efforts will be used to recruit African American and Latino populations, the first and second most common racial/ethnic groups in Prince George’s County. Finally, the PLOT Study will also examine how contextual effects (e.g., neighborhood built environment) impact PT, AT and physical activity.

## 1. Introduction

### 1.1. Obesity and Physical Activity

An issue of great public health concern is that approximately two-thirds of adults and youth in Prince George’s County, Maryland, a suburb of Washington, D.C. comprised predominantly of African Americans, are overweight or obese (overweight/obese), a proportion that exceeds the average in the entire State of Maryland [1,2]. While weight reduction and maintenance is associated with physical activity, only 46% of adults and 35% of youth are achieving Physical Activity Guidelines for Americans (PAGA) within Prince George’s County [1,3,4]. Sociodemographic disparities among this population further compound these public health issues. Within Maryland, the overweight/obesity rate for White adults is 64%, compared to 72% of African American adults [5]. In contrast to 26% of Prince George’s County White adolescent youth, 30% and 39% of African American and Latino youth are overweight/obese, respectively [4]. With further stratification, strikingly more disparate levels of overweight/obese have been recognized when comparing White (63%), African American (82%), and Latina (77%) women, as well as, White (29%), African American (36%), and Latina (37%) girls [6]. Interestingly, trends in the reverse direction are found for physical activity whereby White (55%) adults are more physically active compared to African American (49%) and Latino (51%) adults [7]. Compared to 19% of Prince George’s County White youth, only 15% and 11% of African American and Latino youth achieve the daily PAGA, respectively [4].

### 1.2. Active Transportation

Although activity is most frequently considered within a recreational context, physical activity can be classified into four domains of life describing how people spend their time including active transportation (AT), such as walking, biking or using public transportation (PT) [8]. AT, with PT, is considered a strategic and integral pathway to improving physical activity levels and thus reducing overweight/obesity levels [9,10,11,12,13,14,15,16,17,18,19]. Since travel is essential to daily living, the level of physical activity involved with PT is particularly significant because PT users typically walk or bike on the origin, destination or both sides of their transit stops [9,10,11,12,13,14]. Recommended daily physical activity levels have been shown to be achieved with PT, however the benefits of PT varies by gender and socioeconomic groups whereby females and individuals with low household incomes demonstrated lower rates of PT related walking [20]. Research has also demonstrated that attitudes towards exercise, social interaction, the environment and trip satisfaction are key motivations that influence an individual’s choice of travel model and behavior [21]. For example, individuals who walk for environmental or fitness reasons tend to walk longer distances and are more satisfied with their commutes [21].

For youth, it has also been found that those who engage in AT have better cardiorespiratory and muscular fitness, increased energy expenditure, more favorable body composition and less weight gain [22,23,24,25]. Despite these benefits, it is not well understood how environmental and social factors are associated independently or collectively with the adoption of AT behavior in youth. Some research has shown that built environmental factors (e.g., neighborhood street connectivity, land use, urbanicity) and social factors (e.g., family time constraints, adolescent fear coping, parental risk perceptions) can influence adolescent AT patterns [26,27,28,29,30,31]. Most youth AT research has emphasized walking and cycling. A few studies have examined PT and youth AT, but all of those studies have occurred outside of the U.S. Those studies reported similar physical activity levels among youth in the United Kingdom who were travelling by PT and those who were walking or cycling [32], a 3% increase in energy expenditure when Australian adolescents changed from car travel to PT [25], a lack of PT being associated with lower rates of walking and cycling among Australian girls [33], and a positive association between PT use and age in Swedish adolescents [34].

Recent AT research has found that the use of PT was associated with a beneficial physical activity outcome for individuals who began to use a new extended light rail introduced in Salt Lake City, Utah [35]. Similarly in King County, Washington, PT users of a new light rail system were found to have more daily overall physical activity and more total walking compared to those who did not use PT [26]. While prior research has demonstrated that AT is associated with physical activity, there are still research gaps in understanding how contextual effects ((a) neighborhood built environment; (b) sociodemographics; and (c) “sense of community”) impact PT use, AT behaviors and attitudes among both adults and youth. Additionally, there is a significant gap in understanding how AT and specifically PT promotes physical activity using a longitudinal research framework at the neighborhood level [36,37].

### 1.3. Sense of Community

“Sense of community”, which leads residents to perceive and associate a strong identity or character with a particular physical setting, is a guiding principle in designing sustainable and livable built environments [36,37]. Likewise, modes of access that allow more direct physical engagement with community, such as with AT, further contribute to developing a “sense of community”. Some research has demonstrated that built environment factors (e.g., neighborhood street connectivity, land use, urbanicity) and sociodemographics (e.g., race/ethnicity, income) can influence AT behaviors and attitudes [26,27,28,29,30,31,38]. However, the dynamic relationship of these contextual effects, along with “sense of community”, has not been extensively explored, especially in a regionally diverse area such as Washington, D.C.

Despite the Washington, D.C. metropolitan area having the second highest PT ridership among all U.S. rapid transit systems, there are a lack of data from controlled, long-term studies in this region [39,40]. Utilizing an expansion of the Washington, D.C. metropolitan area transportation system with a new light rail line, the Purple Line Outcomes on Transportation (PLOT) Study will address these research gaps. The PLOT Study, a prospective, longitudinal, natural experiment, will investigate how changes to transportation infrastructure impact PT use, AT behaviors and attitudes and physical activity among adults and youth within Prince George’s County. While examining the entire Prince George’s County population residing within the Purple Line environment, this study will also focus on American and Latino populations disproportionally impacted by physical inactivity, obesity, and other obesity-related comorbidities [41,42,43,44].

### 1.4. Natural Experiment

Even though African Americans and Latinos are both disproportionally impacted by the public health issues of overweight/obesity and physical inactivity, these populations are significantly underrepresented in research studies examining these health issues due to a strong hesitancy and an overarching sense of distrust of the medical and research establishment [41,42,44]. Inadequate recruitment efforts to target African American and Latino participants have been made by study investigators and the National Institutes of Health (NIH) have noted that “natural experiments can allow insights into the effects that programs, interventions, or policies have on health-related outcomes…on low-income and at-risk populations” [41].

A new opportunity for AT in Prince George’s County leverages on the forthcoming Purple Line. Anticipated to begin operation in 2022 under the leadership of the Maryland Transit Administration (MTA), this proposed 16-mile light rail line with 11 stops/stations in Prince George’s County will potentially provide a new opportunity for Prince George’s County residents to engage in AT on their way to many destinations [45]. The PLOT Study will take advantage of this natural experiment in Prince George’s County, an area enduring significantly pronounced racial/ethnic and gender-based disparities of adult and youth overweight/obesity and physical activity rates [1,2]. Natural experiments have emerged as research designs that provide rigorous evidence demonstrating the influence of the built environment on physical activity. In its own novel and timely way, the introduction of the Purple Line, an “intervention” will serve as a built environment modification for Prince George’s County residents. While similar natural experiment studies examining the impact of a newly introduced or expanded transportation infrastructure within neighborhood communities on adult AT have been conducted in other U.S. cities, those studies lacked diverse samples with African American and Latino populations, who have not only been historically underrepresented, but also underrepresented in research studies examining important public health issues [16,26,35,46,47,48]. Overall these studies found positive associations between AT and the presence of a new neighborhood transit option. Specifically, Miller et al. extended upon the Brown et al. 2015 quasi-experimental study, which assessed effects on physical activity and weight among participants in a complete street intervention that extended a light-rail line in Salt Lake City, Utah [35,48]. With the use of individual global positioning system (GPS) and accelerometer data, this study, consisting predominately of White participants, found that transit use directly generated new physical activity, thus yielding beneficial physical activity and body mass index outcomes [35,48]. Research on transportation-oriented developments (TOD), or rather mixed use community developments with walkable neighborhoods and located within walking distance to quality public transportation, have also examined the impact of TODs on physical activity. For example, one TOD cross-sectional study found that approximately 20% of TOD residents achieved their recommended levels of physical activity exclusively through travelling to work or school and to their preferred grocery store [49]. Although this particular study was not a natural experiment, the findings were significant and relevant to the PLOT Study. Pointedly, telecommuting or teleworking (e.g., not traveling to work), a very common work model within the Washington, D.C. metropolitan area, was identified as a detrimental influence of AT and physical activity [49].

The PLOT Study will specifically recruit participants from African American and Latino populations, the first (65%) and second (17.8%) most common racial/ethnic groups in Prince George’s County [50], who are (a) disproportionally impacted by physical inactivity related health outcomes and (b) underrepresented historically, as well as, in research studies. Furthermore, there is a paucity of research on youth PT and AT in the U.S. [25,32,33,51]. To the best of our knowledge, the PLOT Study will be the first study to focus specifically on the impact of newly introduced light rail on both adult and youth AT within the U.S. and specifically within the Washington, D.C. metropolitan area. Overall, the objective of the PLOT Study is to examine pre- and post-Purple Line PT use, AT behaviors and attitudes and physical activity and how contextual effects impact these activity variables with a diverse and historically underrepresented population. 

### 1.5. Conceptual Model

**Figure 1:**
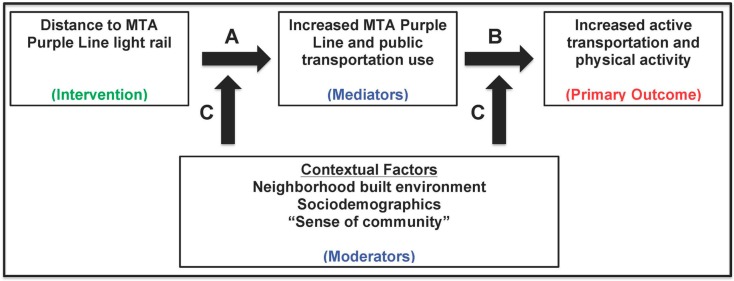
PLOT Study Conceptual Model.

A conceptual model of how the forthcoming Purple Line may affect PT use, AT and physical activity is illustrated in Figure 1 [52]. PLOT Study researchers will examine how access to the Purple Line affects PT (Pathway A) and then how this behavior affects AT behaviors and physical activity (Pathway B). Moreover, the contextualization of these behavior changes through the interaction or moderating effects of neighborhood built environment; sociodemographic; and “sense of community” variables (Pathway C) will also be explored. The PLOT Study will examine the pathways in this conceptual model during the pre- and post-Purple Line periods while also gaining knowledge of the most appropriate strategies for research engagement of underrepresented African American and Latino populations.

## 2. Materials and Methods

### 2.1. Pre-Purple Line Period

#### 2.1.1. Design and Sample

Using an exploratory sequential mixed-methodology design during the baseline pre-Purple Line period, the PLOT Study will occur in two phases (qualitative phase + quantitative phase). Phase I (qualitative) will occur in December 2017–March 2018. Within Phase II (quantitative), a rolling recruitment and enrollment strategy involving three questionnaire deployment pathways will be employed from April 2018 to December 2021 in order to achieve a desired baseline cohort size [53]. The first year of Phase II (quantitative) will occur in two waves (1st Wave: April 2018–June 2018; 2nd Wave: September 2018–November 2018). Subsequent years during Phase II will continue to entail a rolling recruitment and enrollment strategy through December 2021.

Phase I focus group, as well as Phase II questionnaire, accelerometry, and travel diary data will be collected to evaluate PT use, AT behaviors and attitudes and physical activity from Prince George’s County adults (18 years and older) and youth (12–17 years) residing in the forthcoming Purple Line environment. In order to effectively evaluate PT use, AT behaviors and attitudes and physical activity among this Prince George’s County population, targeted efforts will be used to recruit African American and Latino participants. These efforts include holding safe-space moderated discussions through focus groups at organizations with an invested interest in the health and well-being of African Americans and Latinos in addition to attending community events, distributing flyers and publishing announcements in areas or communication materials which are heavily utilized by these targeted populations.

#### 2.1.2. Focus Groups (Phase I)—Adults

Focus groups will be used at the commencement of the study during the first phase (qualitative) in order to gain knowledge of the most appropriate strategies for research engagement of underrepresented African American and Latino populations disproportionally impacted by the aforementioned adverse health outcomes. Along with a lack of willingness and an overarching sense of distrust stemming from historical injustices, these populations have been underrepresented in research studies, particularly within other similar natural experiment studies involving the examination of the built environment [41,42,43,44]. Partnerships with trusted organizations, such as CASA de Maryland and local churches, along with the endorsement of PLOT Study participants throughout the focus group phase, will fortify community relationships and build trust between the community and researchers. Therefore, this initial qualitative data collection inquiry on research engagement methodologies relevant to PLOT Study research objectives will further inform the development and implementation of the PLOT Study second (quantitative) phase.

Four focus groups will be conducted in English and/or Spanish to Prince George’s County African American and Latino adults. With existing community partnerships, participants will be recruited. Specifically, CASA de Maryland will dissemination PLOT Study information to its community members, provide meeting spaces for participants, and facilitate focus groups in the Spanish language. Moreover, PLOT Study researchers will meet with church leaders in Prince George’s County to recruit interested parishioners for focus group participation. Recruitment announcements will be provided to parishioners through the church leaders, in the church bulletins, and/or during church services by the PLOT Study’s Principal Investigator. Using a semi-structured focus group guide, participants in each focus group will be asked questions to generate a discussion on their attitudes and perceptions of participating in research studies. In addition, participants will also have the opportunity to share their attitudes and perspectives on the forthcoming Purple Line; their current neighborhood built environment and how it may change with the new Purple Line; and their “sense of community” within their neighborhood. Probes and clarifying questions will be used as needed to expound on individual experiences and ideas. All focus groups will take place at CASA de Maryland, Susan S. Mona Center for Health and Wellness and local churches. Each focus group will take approximately one hour and all participants will receive $25 dollars for participation. An analysis and understanding of focus group data will custom tailor the most appropriate recruitment strategies for capturing a diverse and representative Prince George’s County sample for the following quantitative phase.

#### 2.1.3. Questionnaire (Phase II)—Adults and Youth

**Figure 2:**
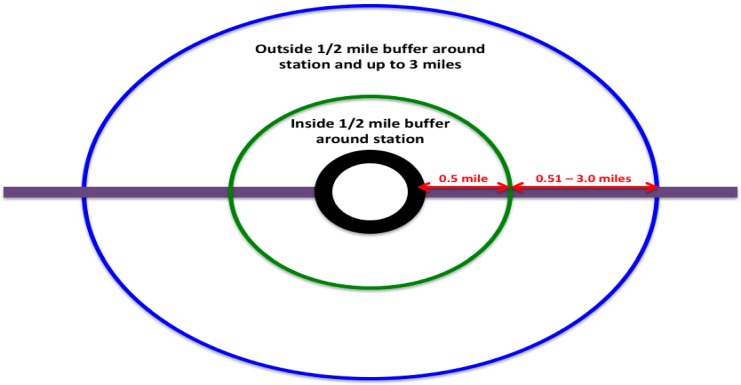
Map of Washington, D.C. metropolitan transportation system and PLOT study area.

**Figure 3:**
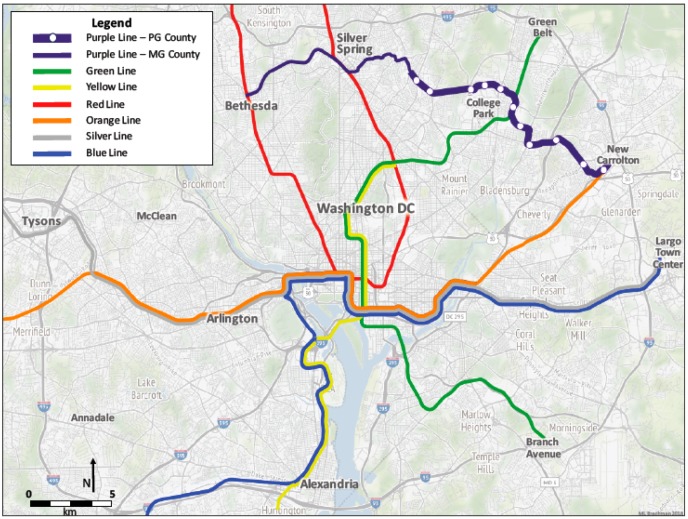
Map of Washington, D.C. metropolitan transportation system and PLOT study area.

Within the second (quantitative) phase, a rolling recruitment and enrollment strategy involving three questionnaire deployment pathways will be employed from January 2018 to December 2021. During the first year of the quantitative phase, an online questionnaire will be deployed in spring/summer (April 2018–June 2018) and fall/winter (September 2018–November 2018) waves to adults (18 years and older) and youth (12–17 years). Subsequent years during this second (quantitative) phase will continue to entail a rolling recruitment and enrollment strategy through December 2021. Physical activity, including PT use, AT behaviors and attitudes, perceived neighborhood characteristics, “sense of community” and sociodemographics data will be collected from participants living in “case” neighborhoods (inside a ½-mile circular buffer around the 11 proposed Prince George’s County Purple Line stations/stops) intended to receive the “intervention” or Purple Line transit service enhancement and from participants in “control” neighborhoods (outside a ½-mile circular buffer and up to three miles around the 11 proposed Prince George’s County Purple Line stations/stops) not intended to receive Purple Line transit service enhancements. Figure 2 illustrates the buffer separating the “case” and “control” neighborhoods while Figure 3 represents the entire Washington, D.C. metropolitan transportation system emphasizing the Prince George’s County Purple Line stations/stops within the PLOT Study catchment area. Using recruitment knowledge learned from the qualitative phase focus groups, participants will be recruited for this second (quantitative) phase.

The first questionnaire deployment pathway will occur through community partnerships, snowball sampling, referrals from existing participants, and mining community email databases (e.g., CASA de Maryland, local churches). Targeted community outreach efforts, such as attending community events (e.g., civic association meetings, church services, farmer’s markets), distributing flyers to recreational community centers and publishing announcements in local circulars with the PLOT Study website and questionnaire link will also be employed to recruit a representative sample and target underrepresented populations. As a second questionnaire deployment pathway, PLOT Study researchers will attend community events equipped with iPads for participants to complete in person. Finally, 11,000 household email addresses with Prince George’s County adults, who also parent a youth, residing inside or outside a ½-mile circular buffer around the 11 proposed Prince George’s County Purple Line stations/stops will be purchased from Alesco Data Group, a direct marketing services company. Through this third deployment, half (*n* = 5500) of the email addresses will recruit residents inside the ½-mile circular buffer and the other half (*n* = 5500) will recruit residents outside the ½-mile circular buffer and up to three miles (Figure 2). Using Qualtrics.com, questionnaires in the English and Spanish language will be deployed. PLOT Study researchers with assistance from CASA de Maryland will perform the questionnaire Spanish translation and pilot test validation. Implicit informed consent will be obtained from parental adults for questionnaire completion. Upon parent email completion of questionnaires, parents will be asked to give consent online for their youth to complete the questionnaire. At community events, where parents and youth are present, written consent from parents will be obtained for youths. If granted, another questionnaire email link will be sent to the youth’s email to complete and assent will be obtained from all youth participants. Fifty participants will be afforded an opportunity to win a $50 gift with a lottery facilitated through Qualtrics.com. With this third email deployment recruitment method, a very conservative 10% response rate based on prior research within this regional population is anticipated [54,55]. However, this method will be supplemented and supported by the previously described community-based recruitment and questionnaire deployment methods. Thus, with four cycles of data collection ((1) pre-Purple Line baseline; (2) 12 months post-Purple Line; (3) 24 months post-Purple Line; (4) 36 months post-Purple Line) and an assumption of 9% participant attrition per year, a sample size of approximately 1000 participants at baseline is anticipated [52].

#### 2.1.4. Accelerometry and Travel Diaries (Phase II)—Adults

Initially, a portion (*n* = 80) of adult participants, will wear an Actigraph**™** accelerometer [56] and GlobalSat DG-100 GPS data logger [57] on the right hip with a dedicated waistband for a seven-day collection period. With additional research funding, this portion of accelerometry participants will be increased during this pre-Purple Line baseline data collection period. This subset of participants, recruited from the questionnaire sample, will also be asked to complete a 7-day travel and activity diary of their activity locations over the accelerometry data collection period. Participants will be asked to record the times when he/she puts on the accelerometer in the morning, takes the accelerometer off at night and any period when the accelerometer is removed for 15 min or longer within their 7-day collection period. The GPS data loggers will record the traveling speed and location of participants at preset times and date intervals. All participants will receive an accelerometer data collection package, which will include one accelerometer and one GPS data logger, as well as, (1) detailed written instructions; (2) a list of frequently asked questions; (3) a link to an instructional YouTube video; (4) a 7-day travel and activity diary log form; (5) a participant checklist for returning the accelerometer, GPS data logger, 7-day travel and activity diary and other study items; and (6) a pre-paid, addressed padded envelope for the return of all data collection materials [52]. The 7-day travel and activity diary log form will collect information on places traveled, times of arrival and departure from each place, modes of travel, and brief information of place activity. Participants will receive their accelerometer data collection packages and informational training by PLOT Study team members at local Prince George’s County recreational or community centers, including the Susan S. Mona Center for Health and Wellness. On these participant training days, the accelerometers will be initialized to begin data collection at midnight and continue collecting data until PLOT Study researchers download the accelerometers. Raw accelerometer data will be sampled at 40 Hz and reintegrated prior to further processing [52]. Over the collection period, two phone calls or text messages will be sent to participants to reinforce instructions and serve as reminders to wear the accelerometer and GPS data logger. Additional phone calls will be made to participants after the 7-day collection period and again in 14 days after the expected return time of the package. If the package is still not received, PLOT Study team members will telephone the participant weekly for a month to request the data collection package and troubleshoot as needed. All accelerometry data participants will be provided a $50 gift.

#### 2.1.5. Field-Based Audits (Phase II)—Purple Line Neighborhoods

Field-based neighborhood audits will be conducted to capture micro-scale built environmental features among street segments that will be in close proximity to the 11 proposed Prince George’s County Purple Line stations/stops. The Analytic Audit Tool will be used to fully explore the relationship between street-scale environments and physical activity [58]. Street segments will be selected using the location of each Prince George’s County Purple Line stations/stops as the center point for a ½-mile network buffer around the station whereby a minimum of 10 census blocks will be selected. Most census blocks have four street segments bounding the census block, which will be captured in the audit [52]. A group of 10–12 auditors will be trained on the use of the Analytic Audit Tool using a customized training manual developed by the PLOT Study team. The audit will be completed on an iPad or on paper and then transcribed into Qualtrics.com. Teams of at least two people per street segment will conduct the audits. Inter- and intra-observer reliability testing will be conducted to assess the percentage of agreement within and between auditors.

### 2.2. Post-Purple Line Period

During the post-Purple Line period, all of the aforementioned research methodologies will be replicated for multiple post-Purple Line study measurements. These post-Purple Line study measurements will occur within one, two and three years of the Purple Line opening. As baseline data is continued to be collected during the pre-Purple Line period, researcher-to-participant contact will be maintained with reminder and update emails of the PLOT Study as well as any construction, opening date, or service updates of the Purple Line.

One key difference between the PLOT Study periods is that unlike the pre-Purple Line period, the post-Purple Line period will employ a two-phase mixed-methodology design with a quantitative phase (e.g., questionnaire, accelerometry, and travel diary data) followed by a qualitative phase (e.g., focus group data). The first phase will occur on a rolling basis throughout the year with focus groups taking place every three months. Different from the pre-Purple Line period, the objective of these focus groups is to elucidate and contextualize the findings from the quantitative phase in addition to understanding the motivating forces of post-Purple Line participant retention. 

### 2.3. Analytic Plan

Focus group data will be digitally recorded, transcribed verbatim, manually coded, and summarized with NVivo 11 [59] using a content analysis technique identifying emergent themes or trends from transcripts. Coding procedures follows a series of steps which include: (1) developing a preliminary set of codes (themes) corresponding to focus group guide; (2) creating additional codes that arise and are of special interest; (3) developing non-substantive codes (e.g., illustrations of particular points); and (4) producing subsequent detailed codes to use for analyses of specific topics. NVivo 11 enables pairs of coders to determine the degree of agreement across focus groups per theme and for the calculation of inter-rater reliability coefficients.

Questionnaire data including self-reported physical activity, PT use, AT behaviors and attitudes, perceived neighborhood characteristics, “sense of community” and sociodemographics will be analyzed using descriptive statistics. Physical activity will be estimated with both questionnaire and accelerometry data. Questionnaire physical activity data will be analyzed by estimating the frequency, duration, intensity, location, and type of physical activity performed. Data downloaded from the accelerometers and GPS data loggers will analyze transportation modes at the trip level (number of steps taken, energy expended). GPS data logger recorded points will be exported using Globalsync v 2.0 [57] to map the routes traveled by participants. Accelerometer data will be screened for periods of wear using established wear-time algorithms [60]. For example, non-wear time will be defined as 90 consecutive minutes of zero counts [60]. All accelerometer estimates will be derived using the vertical axis data and total accelerometer counts per day (TAC/day) will be calculated using summed daily counts detected over wear periods. Minutes per day in different intensity levels will be estimated using substantiated count threshold values of intensity (sedentary (0–99 counts per minute (CPM)); light (100–1951 CPM); moderate (1952–5724 CPM); vigorous (≥5725 CPM)) [61]. Estimated time spent in moderate-to-vigorous physical activity (MVPA) per day will be calculated using a ≥1952 CPM benchmark. Finally, the summary estimates will be computed by averaging daily estimates across total number of days worn for all participants with ≥4 of the 7 days and ≥10 h per day of valid wear time.

Specific research hypotheses will be tested using latent growth curve (LGC) models. This highly flexible technique can model linear and curvilinear relationships and incorporate other statistics to determine if the hypothesized models adequately fit the observed data [52,62]. Additionally, full information maximum likelihood (FIML) estimation will be used so that incomplete participant data can be analyzed [52]. To understand the full mediation model represented by Pathway C in the PLOT Study Conceptual Model (Figure 1), a parallel process growth curve model will be constructed. This will enable testing of the causal chain hypothesis: Purple Line access → PT and AT behavior → physical activity. These LGC models will also allow for participant clustering around Purple Line stations or within neighborhoods, census tracts, and zip codes. Model extension can also accommodate for an examination of demographic, social, or built environment variable interactions. In addition to these analyses, multilevel generalized linear mixed-effects models (GLMM) will be fitted for cross-sectional data analysis. GLMM, an expansion of the general linear model, including both fixed and random effects, will test research hypotheses inferred from estimated risk ratios.

The field-based neighborhood audits will capture micro-scale built environmental features. Macro-scale built environment features will be assessed with Geographic Information Systems (GIS) mapping and spatial analyses. The spatial definition of neighborhood has not been explicitly defined, however, there are several commonly used spatial unit definitions in the built environment research field [52,63]. To further explore the question of whether PT use and AT behaviors and physical activity among the participants with access to the Purple Line interact with built environment features, a series of built environment variables using GIS will be constructed using an administrative boundary approach (e.g., census tracks) and an individualized participant approach (e.g., street network buffers) [52]. Participant home addresses and frequently reported destinations obtained from the 7-day travel and activity diaries will be geocoded and spatially linked to the administrative boundaries in order to construct network buffers of varying radii (e.g., 500, 1000, and 1500 m), thus generating individual participant spatial neighborhood units. Finally, built environment variables found to be associated with physical activity behaviors, such as residential density, street connectivity, land use mix, park density, tree canopy coverage, public transit stop density, sidewalk length and coverage, and bicycle lane length and coverage will be constructed using ArcGIS software [64,65,66].

### 2.4. Ethics

The Institutional Review Board at The University of Maryland at College Park approved this study protocol (UMCP, 1132306-1). For the focus group phase of the study, all adult participants will provide written consent. Information about the PLOT Study will be provided at the beginning of the questionnaire. This information will be written at a reading level that is easily understood by all, indicating that participation is voluntary, that he/she is free to withdraw participation any time without penalty, a description of measures that will be taken to ensure privacy, and how the results will be used. Adult participants will be required to click a button to acknowledge that they have read the study information and then informed consent will be obtained for questionnaire completion. The informed consent form will be returned electronically with the questionnaire. Participants will be instructed to print or email a copy for their records. Upon completion of questionnaires, adults of youth will be asked to give consent for their youth to complete the same questionnaire. For consented youth, questionnaire will be sent to the youth by email. Youth participants will be required to click a button to acknowledge that they have read the study information and then informed consent will be obtained for questionnaire completion. Again, participants will be instructed to print or email a copy for their records. Accelerometry participants will sign written consent forms when they receive their accelerometer data collection packages and informational training by PLOT Study team members at local Prince George’s County recreational or community centers. 

## 3. Discussion

A unique facet of this natural experiment study is the diversity of the Prince George’s County Study population. The PLOT Study will have the ability to pull from a population that consist of more than 75% African American and Latino residents in Prince George’s County and as described targeted efforts will be employed to recruit participants from these populations [50]. Prior natural experiment studies conducted in King County, Washington, Salt Lake City, Utah and Charlotte, North Carolina consisted of over 70% White and over 70% non-Hispanic participants [16,26,46]. The inclusion of these underrepresented populations is crucial to the validity of the study results, but more importantly the adequate representation of the PLOT Study is essential to address the research questions and policy issues that are specifically tailored to Prince George’s County.

Another singular characteristics of the PLOT Study is the incorporation of youth as there is a paucity of research on youth PT and AT in the U.S. [25,32,33,51]. PLOT is a pioneering study focusing specifically on the impact of newly introduced light rail on both adult and youth AT within the U.S. and specifically within the Washington, D.C. metropolitan area. The PLOT Study will also improve our understanding of the relationships between the built and social environments and PT, AT, and physical activity while also adding a new and unexplored knowledge base of this research among adults and youth. Subjective variables, such as built environment and neighborhood perception or “sense of community”, and influences by cultural, ethnic or other individual characteristics may also have a significant impact on PT and AT behaviors and thus overall physical activity. Therefore, this research will provide additional insight to the bi-directional connection and dependence of individual subjective choices, objective built environment measures, and PT and AT behaviors in adults and youth.

While the primary challenge in the PLOT Study is recruiting participants, the goal of recruiting a diverse and representative Prince George’s County sample is attainable given the mixed-methodology design of the study and the multi-layered recruitment efforts. Another challenge involves the discomfort of the participants due to a variety of reasons (e.g., English as a second language; undocumented status). Employing measures to heavily involve our community partners (e.g., focus groups held at locations of familiarity) will be aimed to ease participant anxiety. A weakness and strength with the PLOT Study relates to the issue of timing. The Purple Line is not scheduled to begin operation until 2022 and this will allow for adequate time to collect pre-Purple Line data. However, an inherent challenge of natural experiments is the accurate timing of data collection completion with an unpredictable and frequently changing intervention timetable that is completely outside of the researcher’s control [67,68]. Legal challenges have delayed the construction of the Purple Line and concerns with transportation induced gentrification as well as the protection of green space have stagnated the support of the forthcoming light rail line [69,70]. Although data collection cannot be delayed indefinitely to accommodate construction or legal delays, the flexibility of funding bodies is essential to adjust for these types of events and situations.

Finally, by illustrating the relationship between a transportation infrastructure change with PT use, AT behaviors and attitudes, physical activity, and contextual effects, local, regional, and national policy and land use planners may find utility with the PLOT Study research findings and thwart the possibility of negative side effects (e.g., transportation induced gentrification). Therefore, engagement of policy and land use planners is critical throughout all study milestones and promulgation of the PLOT Study findings to all interested and influential parties will augment the potential impact of these findings on a much larger scale. 

## 4. Conclusions

The methodology of PLOT, a quasi-experimental longitudinal pre-post study, has been described. This natural experiment study intends to measure the impact of a transportation infrastructure change on PT use, AT behaviors and attitudes and physical activity among adults and youth, while also assessing the influence of neighborhood built environment, sociodemographics, and “sense of community” contextual effects.

## References

[1] Prince George’s County Health Department Health Report 2015. http://www.princegeorgescountymd.gov/ArchiveCenter/ViewFile/Item/1557.

[2] Prince George’s County Planning Board A Discussion of Community Health and Food Access in Prince George’s County: Prince George's County General Plan Update. http://planpgc2035.com/sites/default/files/documents/CommunityHealthandFoodAccessPolicyPaper.pdf.

[3] Centers for Disease Control and Prevention Youth Risk Behavior Survey Results—Maryland Middle School—Prince George's County: Physical Activity Summary Tables. http://phpa.dhmh.maryland.gov/ccdpc/Reports/Documents/2014YRBSReports/2014PrinceGeorgeMSSummaryTables.pdf.

[4] Centers for Disease Control and Prevention Youth Risk Behavior Survey Results—Maryland High School—Prince George's County: Physical Activity Summary Tables. http://phpa.dhmh.maryland.gov/ccdpc/Reports/Documents/2014YRBSReports/2014PrinceGeorgeHSSummaryTables.pdf.

[5] U.S. Department of Health and Human Service, Centers for Disease Control and Prevention Behavioral Risk Factor Surveillance System Survey—Prevalence & Trends Data. Maryland Overweight/Obesity. https://www.cdc.gov/brfss/brfssprevalence/index.html.

[6] State of Obesity Special Report: Better Policies for a Healthier America. Racial and Ethnic Disparities in Obesity—An In-Depth Look at the Inequities that Contribute to Higher Obesity Rates in Black and Latino Communities. http://stateofobesity.org/files/stateofobesity2015.pdf.

[7] U.S. Department of Health and Human Service, Centers for Disease Control and Prevention Behavioral Risk Factor Surveillance System Survey—Prevalence & Trends Data. Maryland Physical Activity. https://www.cdc.gov/brfss/brfssprevalence/index.html.

[8] Pratt M., Macera C.A., Sallis J.F., O’Donnell M., Frank L.D. (2004). Economic interventions to promote physical activity: Application of the SLOTH model. Am. J. Prev. Med..

[9] Besser L.M., Dannenberg A.L. (2005). Walking to public transit: Steps to help meet physical activity recommendations. Am. J. Prev. Med..

[10] Lachapelle U. (2015). Walk, Bicycle, and Transit Trips of Transit-Dependent and Choice Riders in the 2009 United States National Household Travel Survey. J. Phys. Act. Health.

[11] Lachapelle U., Frank L.D. (2009). Transit and health: Mode of transport, employer-sponsored public transit pass programs, and physical activity. J. Public Health Policy.

[12] Chaix B., Kestens Y., Duncan S., Merrien C., Thierry B., Pannier B., Brondeel R., Lewin A., Karusisi N., Perchoux C. (2014). Active transportation and public transportation use to achieve physical activity recommendations? A combined GPS, accelerometer, and mobility survey study. Int. J. Behav. Nutr. Phys. Act..

[13] Rissel C., Curac N., Greenaway M., Bauman A. (2012). Physical activity associated with public transport use—A review and modelling of potential benefits. Int. J. Environ. Res. Public Health.

[14] Bopp M., Gayah V.V., Campbell M.E. (2015). Examining the link between public transit use and active commuting. Int. J. Environ. Res. Public Health.

[15] Edwards R.D. (2008). Public transit, obesity, and medical costs: Assessing the magnitudes. Prev. Med..

[16] MacDonald J.M., Stokes R.J., Cohen D.A., Kofner A., Ridgeway G.K. (2010). The effect of light rail transit on body mass index and physical activity. Am. J. Prev. Med..

[17] Villanueva K., Giles-Corti B., McCormack G. (2008). Achieving 10,000 steps: A comparison of public transport users and drivers in a university setting. Prev. Med..

[18] Lachapelle U., Frank L., Saelens B.E., Sallis J.F., Conway T.L. (2011). Commuting by public transit and physical activity: Where you live, where you work, and how you get there. J. Phys. Act. Health.

[19] Stokes R.J., MacDonald J., Ridgeway G. (2008). Estimating the effects of light rail transit on health care costs. Health Place.

[20] Wasfi R.A., Ross N.A., El-Geneidy A.M. (2013). Achieving recommended daily physical activity levels through commuting by public transportation: Unpacking individual and contextual influences. Health Place.

[21] Manaugh K., El-Geneidy A.M. (2013). Does distance matter? Exploring the links among values, motivations, home location, and satisfaction in walking trips. Transp. Res. Part A.

[22] Ostergaard L., Kolle E., Steene-Johannessen J., Anderssen S.A., Andersen L.B. (2013). Cross sectional analysis of the association between mode of school transportation and physical fitness in children and adolescents. Int. J. Behav. Nutr. Phys. Act..

[23] Drenowatz C., Kobel S., Kettner S., Kesztyus D., Wirt T., Dreyhaupt J., Steinacker J.M. (2013). Correlates of weight gain in German children attending elementary school. Prev. Med..

[24] Andersen L.B., Lawlor D.A., Cooper A.R., Froberg K., Anderssen S.A. (2009). Physical fitness in relation to transport to school in adolescents: The Danish youth and sports study. Scand. J. Med. Sci. Sports.

[25] Booth M.L., Okely A.D., Denney-Wilson E., Hardy L.L., Dobbins T., Wen L.M., Rissel C. (2007). Characteristics of travel to and from school among adolescents in NSW, Australia. J. Paediatr. Child Health.

[26] Saelens B.E., Moudon A.V., Kang B., Hurvitz P.M., Zhou C. (2014). Relation between higher physical activity and public transit use. Am. J. Public Health.

[27] Saelens B.E., Sallis J.F., Frank L.D. (2003). Environmental correlates of walking and cycling: Findings from the transportation, urban design, and planning literatures. Ann. Behav. Med..

[28] Johansson K., Hasselberg M., Laflamme L. (2010). Young adolescents’ independent mobility, related factors and association with transport to school. A cross-sectional study. BMC Public Health.

[29] Trapp G.S., Giles-Corti B., Christian H.E., Bulsara M., Timperio A.F., McCormack G.R., Villaneuva K.P. (2012). Increasing children’s physical activity: Individual, social, and environmental factors associated with walking to and from school. Health Educ. Behav..

[30] Cloutier M.S., Bergeron J., Apparicio P. (2011). Predictors of parental risk perceptions: The case of child pedestrian injuries in school context. Risk Anal..

[31] Miller W.C., Redmond J.G., Vaux-Bjerke A.T. (2013). Activity patterns and perceptions about active transport to school. Am. J. Health Behav..

[32] Owen C.G., Nightingale C.M., Rudnicka A.R., Sluijs E.M., Ekelund U., Cook D.G., Whincup P.H. (2012). Travel to school and physical activity levels in 9–10 year-old UK children of different ethnic origin; Child Heart and Health Study in England (CHASE). PLoS ONE.

[33] Timperio A., Crawford D., Telford A., Salmon J. (2004). Perceptions about the local neighborhood and walking and cycling among children. Prev. Med..

[34] Johansson K., Laflamme L., Hasselberg M. (2012). Active commuting to and from school among Swedish children—A national and regional study. Eur. J. Public Health.

[35] Brown B.B., Werner C.M., Tribby C.P., Miller H.J., Smith K.R. (2015). Transit use, physical activity, and body mass index changes: Objective measures associated with complete street light-rail construction. Am. J. Public Health.

[36] Puddifoot J.E. (1995). Dimensions of community identity. J. Community Appl. Soc. Psychol..

[37] McMillan D.W., Chavis D.M. (1986). Sense of community: A definition and theory. J. Community Psychol..

[38] Sallis J.F., Floyd M.F., Rodriguez D.A., Saelens B.E. (2012). Role of built environments in physical activity, obesity, and cardiovascular disease. Circulation.

[39] UNC School of Government (2014). Let’s Take a Ride: 5 Largest US Public Transit Systems.

[40] APTA (American Public Transportation Association) (2016). Public Transportation Ridership Report—Fourth Quarter 2015.

[41] NIH (National Institutes of Health) Methods for Evaluating Natural Experiments in Obesity. Proceedings of the Pathways to Prevention Workshop, Natcher Conference Center, NIH Campus.

[42] Terrell F., Moseley K.L., Terrell A.S., Nickerson K.J. (2004). The relationship between motivation to volunteer, gender, cultural mistrust, and willingness to donate organs among Blacks. J. Natl. Med. Assoc..

[43] Brandon D.T., Isaac L.A., LaVeist T.A. (2005). The legacy of Tuskegee and trust in medical care: Is Tuskegee responsible for race differences in mistrust of medical care?. J. Natl. Med. Assoc..

[44] Watson B., Robinson D.H., Harker L., Arriola K.R. (2016). The Inclusion of African-American Study Participants in Web-Based Research Studies: Viewpoint. J. Med. Internet Res..

[45] MDDOT (Maryland Department of Transportation) (2016). Fast Facts on the Purple Line Project.

[46] Brown B.B., Werner C.M. (2008). Before and After a New Light Rail Stop: Resident Attitudes, Travel Behavior, and Obesity. J. Am. Plan. Assoc..

[47] Lang R., Kelkar V.A., Byrd J.R., Edwards C.L., Pericak-Vance M., Byrd G.S. (2013). African American participation in health-related research studies: Indicators for effective recruitment. J. Public Health Manag. Pract..

[48] Miller H.J., Tribby C.P., Brown B.B., Smith K.R., Werner C.M., Wolf J., Wilson L., Oliveira M.G. (2015). Public transit generates new physical activity: Evidence from individual GPS and accelerometer data before and after light rail construction in a neighborhood of Salt Lake City, Utah, USA. Health Place.

[49] Langlois M., Wasfi R.A., Ross N.A., El-Geneidy A.M. (2016). Can transit-oriented developments help achieve the recommended weekly level of physical activity?. J. Transp. Health.

[50] U.S. Census (2015). QuickFacts—Prince George’s County, Maryland.

[51] Carver A., Timperio A.F., Hesketh K.D., Ridgers N.D., Salmon J.L., Crawford D.A. (2011). How is active transport associated with children’s and adolescents’ pshysical activity over time?. Int. J. Behav. Nutr. Phys. Act..

[52] Durand C.P., Oluyomi A.O., Gabriel K.P., Salvo D., Sener I.N., Hoelscher D.M., Knell G., Tang X., Porter A.K., Robertson M.C. (2016). The Effect of Light Rail Transit on Physical Activity: Design and Methods of the Travel-Related Activity in Neighborhoods Study. Front. Public Health.

[53] Knell G., Durand C.P., Shuval K., Iii H.W.K., Salvo D., Sener I., Gabriel K.P. (2018). Transit use and physical activity: Findings from the Houston travel-related activity in neighborhoods (TRAIN) study. Prev. Med. Rep..

[54] Roberts J.D., Knight B., Ray R., Saelens B.E. (2016). Parental perceived built environment measures and active play in Washington, DC metropolitan children. Prev. Med. Rep..

[55] Fincham J.E. (2008). Response rates and responsiveness for surveys, standards, and the Journal. Am. J. Pharm. Educ..

[56] ActiGraph (2018). ActiGraph, LLC.

[57] USGlobalSat (2014). GlobalSat DG-100 GPS.

[58] Brownson R.C., Ramirez L.K.B., Hoehner C.M., Cook R.A. (2003). Analytic Audit Tool and Checklist Audit Tool. Active Living Research.

[59] NVivo (2015). NVivo Qualitative Data Analysis Software.

[60] Choi L., Liu Z., Matthews C.E., Buchowski M.S. (2011). Validation of accelerometer wear and nonwear time classification algorithm. Med. Sci. Sports Exerc..

[61] Freedson P.S., Melanson E., Sirard J. (1998). Calibration of the Computer Science and Applications, Inc. accelerometer. Med. Sci. Sports Exerc..

[62] Preacher K.J. (2008). Latent Growth Curve Modeling.

[63] Matthews S.A. (2008). The salience of neighborhood: Some lessons from sociology. Am. J. Prev. Med..

[64] ESRI (1999). ArcGIS Desktop.

[65] Thornton L.E., Pearce J.R., Kavanagh A.M. (2011). Using Geographic Information Systems (GIS) to assess the role of the built environment in influencing obesity: A glossary. Int. J. Behav. Nutr. Phys. Act..

[66] Brownson R.C., Hoehner C.M., Day K., Forsyth A., Sallis J.F. (2009). Measuring the built environment for physical activity: State of the science. Am. J. Prev. Med..

[67] Ogilvie D., Bull F., Cooper A., Rutter H., Adams E., Brand C., Ghali K., Jones T., Mutrie N., Powell J. (2012). Evaluating the travel, physical activity and carbon impacts of a ‘natural experiment’ in the provision of new walking and cycling infrastructure: Methods for the core module of the iConnect study. BMJ Open.

[68] Veitch J., Salmon J., Carver A., Timperio A., Crawford D., Fletcher E., Giles-Corti B. (2014). A natural experiment to examine the impact of park renewal on park-use and park-based physical activity in a disadvantaged neighbourhood: The REVAMP study methods. BMC Public Health.

[69] Smith M. (2017). Md. Transportation Chief: Lawsuits Mean Delay in Purple Line Opening. https://wtop.com/dc-transit/2017/11/md-transportation-chief-lawsuits-mean-delay-purple-line-opening.

[70] Metcalf A. Judge Delays Decision on Purple Line Tree Cutting: Opponents Believe Large Trees Are Wrongfully Being Cut Down. http://www.bethesdamagazine.com/Bethesda-Beat/2017/Judge-Delays-Decision-on-Purple-Line-Tree-Cutting/.

